# Designing Reciprocative Dynamic Linking to improve learners’ Representational Competence in interactive learning environments

**DOI:** 10.1186/s41039-017-0046-8

**Published:** 2017-02-10

**Authors:** Mrinal Patwardhan, Sahana Murthy

**Affiliations:** Inter Disciplinary Program in Educational Technology, Indian Institute of Technology Bombay, Mumbai, 400076 India

**Keywords:** Multiple external representations, Interactive learning environment, Simulation, Representational Competence, Affordance, Cognitive load

## Abstract

Learning from interactive learning environments enriched with multiple external representations (MERs) is often beneficial. The learning benefits of MERs highly rely on the development of Representational Competence. Representational Competence refers to an ability to translate and see relations between MERs. The relevant research findings have consistently reported learners’ difficulty in relating and translating in MERs due to insufficient development of Representational Competence. Although dynamic linking is one of the strategies recommended to address this issue, it offers mixed results. This paper reports design of a new interaction feature that overcomes some of the limitations of traditional dynamically linked representations. We designed an additional interaction in dynamically linked MERs to support learners’ cognitive demands; we refer to this as *Reciprocative Dynamic Linking*. The goal of this additional affordance was to strengthen learners’ cross-representation cognitive linkage by promoting Representational Competence. The paper reports the study conducted to investigate effects of Reciprocative Dynamic Linking on students’ Representational Competence. The said study was conducted in a course on Signals and Systems from Electrical Engineering program (*N* = 24). The subjects were assigned to two conditions: a Simulation and a Simulation with Reciprocative Dynamic Linking. The representation competence was assessed with an instrument for measuring Representational Competence within Signals and Systems domain. The effect of Reciprocative Dynamic Linking on learners’ cognitive load was also investigated. The results confirmed that Reciprocative Dynamic Linking could lead to improvement in Representational Competence and thus, higher learning for “Apply and Analyze Procedural knowledge” categories of tasks. Reciprocative Dynamic Linking also promoted germane cognitive load of learners, as it could offer the required cognitive support to improve learners’ Representational Competence. The findings from semi-structured interviews and screen capture analysis corroborated the results. This paper provides details of how to design Reciprocative Dynamic Linking in interactive learning environments and its effect on learners’ Representational Competence. Apart from establishing learning effectiveness of Reciprocative Dynamic Linking, the study further contributes by confirming the role of cognitive processing of learners while learning from interactive learning environments. The findings from the study suggest designing strategies not for just creating highly interactive learning environments but equipping a given learning environment with conducive interaction features that foster learning.

## Introduction

Various forms of computer-based learning environments prevalent in education include animation, interactive simulation, gaming environment, smart boards, adaptive learning environments, ubiquitous learning environments, and various system simulators. In this paper, we refer “interactive learning environments” (ILEs) to computer-based learning environments; which present content dynamically in an interactive manner, permitting interactions between a learner and a learning material with the help of different kinds of interaction features. Interactive simulation, one of the type of interactive learning environments, have been used in the teaching-learning of elementary level science concepts (Barak et al. 2011), as well as complex concepts or processes in engineering and allied courses (Boucheix and Schneider 2009; Lattu et al. 2003; de los Santos Vidal et al. 1996; Wang et al. 2011). ILEs are often enriched with multiple external representations (MERs) to explain relevant scientific concepts and phenomenon.

Learning with MERs facilitates and strengthens learning process by providing several mutually referring sources of information (Moreno and Durán 2004). ILEs include a variety of multiple representations in the form of audio, videos, animations, tables, and graphs. Learners can integrate concepts from different representation formats and sensory modalities into one meaningful experience (Moreno and Mayer 2007). Using MERs, learners build abstractions that promote deeper understanding of domain (Ainsworth and VanLabeke 2004). The coordination of different representations in a cohesive manner and explicit identification of their relations support students’ understanding at a deeper level.

Research findings related to learning impact of MERs have consistently reported learners’ difficulty in relating and translating in MERs. Representational Competence refers to learner’s ability to “reflectively use a variety of multiple external representations or visualizations, singly and together, to think about, and act on the underlying physical entities and processes in a domain” (Kozma and Russell 1997). This includes an ability to translate and see the relations between representations. The learning benefits of MERs highly rely on how students translate between and connect across multiple external representations (Ainsworth 1999; Kozma and Russell 1997; Wu and Shah 2004). To overcome the learning difficulty in relating and translating in MERs, researchers have recommended support for learners in translation by means of appropriate design features and design guidelines (Tabachneck et al. 1994; Kozma 2003). Dynamic linking (also referred to as dyna-linking) in MERs has been one such popular strategy adopted for enabling translation among representations (Ainsworth 1999). It is expected that dyna-linking helps learners to establish relationships between representations (Kaput 1989; Scaife and Rogers 1996). However, the results of empirical studies have been mixed. Dyna-linking bears the risk of users remaining passive learners due to automatic transitions and learners experiencing more cognitive load due to the requirement of focusing on multiple contents of the learning environment (van der Meij and de Jong 2006). In a comparative study, simply linking representations dynamically did not lead to improved learning compared to non-linking (van der Meij and de Jong 2006). Thus, due to the mixed nature of results, there is a demand for further research to ensure learning benefits from dyna-linked MERs.

Research in cognitive and learning sciences has acknowledged that deeper learning with multiple external representations depends on certain conditions including the cognitive load of learners (Ainsworth 1999, 2006; Kozma and Russell 1997; Wu and Shah 2004). Cognitive load is not simply considered as a by-product of the learning process but as a major factor that determines success of an instructional intervention. Learning is hindered when cognitive overload occurs and working memory capacity is exceeded (de Jong 2010). Learning with separate representations generates a heavy cognitive load leaving fewer resources for actual learning, since learners are required to relate disparate sources of information (Sweller 1989). Thus, instructional control of cognitive load while leaning from ILEs, especially while dealing with features such as MERs, is vital. Therefore, learning environments need to be designed with interactive features that meet learners’ cognitive processing demands.

This article reports the design of a new interaction feature that enhances the learning process by overcoming some limitations of traditional dynamically linked MERs. Considering the need to strengthen learners’ Representational Competence and support their cognitive load demands while translating among MERs, we designed an additional interaction in dynamically linked MERs. We refer to this as *Reciprocative Dynamic Linking* (RDL) which is an affordance to select and manipulate each of the MERs individually in a reciprocative manner. Most conventional dynamically linked MERs permit and offer only one representation for manipulation, and the corresponding changes in other representations are displayed. This does not allow learners to learn the reciprocal linkage of other representations with the manipulated representation. Learners’ attempts to develop this cross-representational linkage by attending multiple representations simultaneously with conventional dynamically linked MERs put additional learning demands on learners (Van der Meij and de Jong, 2006). With Reciprocative Dynamic Linking, learners not just observe dynamic changes of MERs but are also able to select and manipulate all representations one by one. Learners are able to see automatic changes occurring in the second representation when the first representation is manipulated, and they are also able to see the changes occurring in the first representation when they actively manipulate the second representation. More importantly, learners are able to make intentional choices about which representation is to be selected for manipulation. The goal of this additional affordance is to strengthen learners’ cross-representation cognitive linkage by promoting Representational Competence. It is expected that this improvement in the Representational Competence would further help learners in improving learning of domain knowledge.

In this paper, we present the design of Reciprocative Dynamic Linking in ILEs developed for a topic on Signal Representation in the course on “Signals and Systems”; a second year course from Electrical Engineering undergraduate program. The paper reports the study conducted to investigate effect of Reciprocative Dynamic Linking on students’ Representational Competence assessed in the selected domain. The effect of Reciprocative Dynamic Linking on learners’ cognitive load was also investigated as a part of this study, as the proposed feature of Reciprocative Dynamic Linking was expected to offer the required instructional support to meet learners’ cognitive load demands. The results confirmed that Reciprocative Dynamic Linking could lead to higher learning, and it offered the required cognitive support to improve learners’ Representational Competence. The findings from semi-structured interviews and screen capture analysis corroborated the results. This paper provides details of how to design Reciprocative Dynamic Linking in ILEs and how it contributes towards improving learners’ Representational Competence.

## Related work

The main strength of MERs in ILEs lies in the different types of (dynamic) representations that can be included and its ability to combine different representations in one interface. MERs offer several learning benefits. Each representation in MERs can show specific aspects of the domain to be learnt. Different types of representations may be useful for different purposes, as they differ in their representational and computational efficiency (Larkin and Simon 1987). Teaching and learning with more representations facilitates and strengthens the learning process by providing several mutually referring sources of information (Kozma and Russell 1997; Grouws 1992) and leads to deeper learning. It has been reported that “*the cognitive linking of representations creates a whole that is more than the sum of its parts … It enables us to ‘see’ complex ideas in a new way and apply them more effectively*” (Kaput 1989, 1992). As reported in the research studies, students generally benefit from being exposed to a wide range of representations and perspectives on a problem, including underlying mathematical and scientific laws, engineering design strategies and objects, as well as the social context (Nathan et al. 2011, 2013; Walkington et al. 2011). It also further highlighted that the coordination of different representations in a cohesive manner and explicit identification of their relations supports student understanding.

Learning from multiple representations is characterized by means of three key functions: (i) to provide complementary information and processes, (ii) to constrain interpretations, and (iii) to construct a deeper domain understanding (Ainsworth 1999). The major learning demands from MERs on learners are to understand the semantics of each representation, to understand which parts of the domain are represented, to relate the representations to each other, and to translate between the representations.

In general, learners’ ability in translating between, seeing the relations between MERs and connecting across MERs, plays an important role in deciding learning effectiveness while learning from MERs (Ainsworth 1999, 2006; Kozma and Russell 1997). The Representational Competence of learners subsumes learners’ abilities to comprehend how two representations are related and how they can be used together. Thus, Representational Competence influences learning from MERs.

When learning with separate representations, learners are required to relate separate sources of information, which may generate a heavy cognitive demand, leaving fewer resources for actual learning, especially in dynamically changing MERs. Thus, learning from MERs places demands on working memory and creates challenges for learners (van Someren et al. 1998), especially those with low prior knowledge (Kozma and Russell 1997; Yerushalmy 1989). These challenges can cause students to interact with simulations randomly, instead of systematically (de Jong and van Joolingen 1998). Such learning limitations affect learners’ understanding and results into a discourse that is constrained by the surface features of individual representations. Thus, the unique cognitive demand while learning MERs is to understand how to translate between representations in dynamic learning environments.

Researchers recommended support for learners in this translation through appropriate design features and design guidelines (Tabachneck et al. 1994; Kozma 2003). A variety of approaches in the form of guidelines such as implicit cues, integrated representations, static linking, dynamic linking, and explicit instruction have been suggested to address students’ such difficulties (Ainsworth 2006; van der Meij and de Jong 2004). While Kozma (Kozma 2003) suggested design principles to increase connections between representations for supporting students’ domain understanding, DeFT (Design, Functions, Tasks) principles were implemented in the DEMIST learning environment (Ainsworth and VanLabeke 2004). These principles recommended dynamical linking (dyna-linking) in MERs, when multiple external representations are used to support complementary roles and information, and to constrain interpretation.

Dyna-linking of MERs has been one such popular strategy for enabling translations between representations (Ainsworth 1999). With dynamically linked representations, actions performed on one representation are automatically shown in all other representations. It is expected that dyna-linking helps learners to establish relationships between representations (Kaput 1989; Scaife and Rogers 1996). It helps learners in accomplishing an important task of translating between representations. Two important learning requirements are considered while designing dyna-linked MERs; the need to learn content from complementary representations and the need to reduce cognitive load of making mental connections between representations (Wu and Puntambekar 2012). The learning benefits from dyna-linked MERs are attributed to the cognitive support extended to learner. As the translations between MERs are taken care by the technology in the learning environment, learners are freed to concentrate on interpreting the representations and their consequences. The Cognitive Theory of Multimedia Learning (Mayer 2001) and the dual channel assumption of Dual Coding Theory (Paivio 1986) support the use of dynamic linking of MERs to reduce the cognitive load upon learners.

An environment using multiple dynamically linked representations can facilitate novices’ learning (Kozma and Russell 1997). While the simultaneously changing representations in dynamic linking have been conceived as a useful feature, it has also received criticism. Ainsworth (1999) cautioned that dynamic linking might leave a learner too passive in the learning process. Dynamic linking may discourage reflection on the nature of the translations, leading to a failure of learner in constructing the required understanding. Another problem with dynamic linking has been that with multiple dynamically changing representations, learners need to attend to changes that occur simultaneously in different regions of various representations, leading to cognitive overload (Lowe 2003). It must be noted that on the one side, while the feature of dyna-linking is being reported to offer cognitive support while learning from MERs, on the other hand, it has also been considered to induce more cognitive load due to the need to attend to changes that occur simultaneously in different regions of various representations. Empirical studies such as one with the SIMQUEST environment (van der Meij and de Jong 2006) found that simply, linking representations dynamically could not improve learning compared with non-linking. It showed some improvement in the learning only with spatially integrated linked representations. Such an integration of representations is not always possible, due to the nature of the learning materials or specific learning goals.

Thus, the nature of results of dyna-linking in MERs is mixed, and hence, there is a need for further research to design dynamic linking with apt interactivity that would offer the necessary cognitive support to learners while translating among representations. This also highlights the relevance of the concept of cognitive load and cognitive load theory while learning with dyna-linked interactive learning environments. The basic idea of cognitive load theory is that cognitive capacity in working memory is limited; so that if a learning task requires too much capacity, learning will be hampered. The recommended remedy is to design instructional systems and features that optimize the use of working memory capacity and avoid cognitive overload.

DeLeeuw and Mayer (2008) theorize that there are three types of cognitive processing (essential, extraneous, and generative) and place them in the *triarchic model of cognitive load*. Mayer proposed this model for organizing framework for the cognitive theory of multimedia learning and stated that a major goal of multimedia learning and instruction is to *“manage essential processing, reduce extraneous processing and foster generative processing”* (Mayer 2009). Intrinsic cognitive load occurs during the interaction between the nature of the material being learnt and the expertise of the learner. The second type, extraneous cognitive load, is caused by factors that aren’t central to the material to be learnt, such as presentation methods or activities that split attention between multiple sources of information, and these should be minimized as much as possible. The third type of cognitive load, germane cognitive load, enhances learning and results in task resources being devoted to schema acquisition and automation. Intrinsic cognitive load cannot be manipulated, but extraneous and germane cognitive loads can be manipulated. As germane cognitive load relates to learner’s engagement in cognitive processing such as mentally organizing the material and relating it to prior knowledge, it is important to channelize and design instructional design strategies to increase germane cognitive load. Thus, we consider designing interaction features to increase germane cognitive load as one of the strategies to offer necessary cognitive support to learners for optimizing cognitive resources while translating among the representations.

On this backdrop, aptly designed interactive features in interactive learning environments can offer the necessary cognitive support to learners. Such features can ensure effective learning from dynamically linked MERs in technology-enhanced learning (TEL) environments. We designed “Reciprocative Dynamic Linking”; an additional interaction feature to offer the required cognitive support to learners while learning from MERs. The following section explains designing of “Reciprocative Dynamic Linking.”

## Designing “Reciprocative Dynamic Linking” in MERs

The goal of Reciprocative Dynamic Linking is to promote Representational Competence by strengthening learners’ cross-representation cognitive linkage while learning from multiple external representations. There have been numerous taxonomies used for defining and categorizing various forms of MERs. We refer to the most commonly used taxonomy (Wu and Puntambekar 2012) that includes four major types of representations: verbal-textual, symbolic-mathematical, visual-graphical, and actional-operational. Considering the domain requirement, symbolic-mathematical and visual-graphical types of MERs are used as forms of multiple representations in the learning materials developed. The key feature of Reciprocative Dynamic Linking has been the reciprocative interface. It allows selection and manipulation of each of the MERs individually in a reciprocative manner. The reciprocative interface is two-way manipulative, and it enables learners to carry out meaningful switchover among MERs resulting in comprehension of relations between them. The dynamically linked MERs can be selected for manipulation using an interactive selection affordance.

The Reciprocative Dynamic Linking derives its base from contemporary theories of cognition such as distributed and embodied cognition (Glenberg et al. 2013). These theories postulate that external representations play more roles than merely decreasing cognitive load and can support operations that are difficult to do by imagination alone (Kirsh 2010). Actions like manipulations could be a way of promoting integration of MERs (Chandrasekharan 2009).

**Fig. 1:**
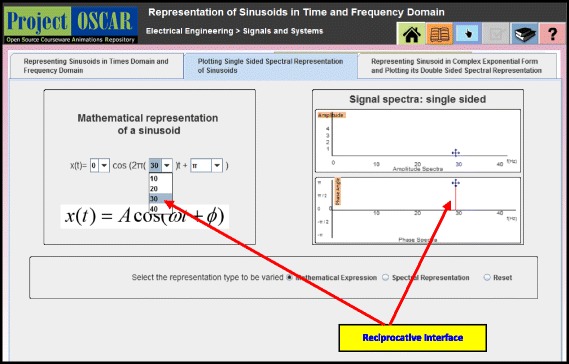
Reciprocative interface of Reciprocative Dynamic Linking

Figure 1 shows the screenshot of a simulation in the topic of signal representation designed with Reciprocative Dynamic Linking. As shown in the Fig. 1, there are two dynamically linked MERs in the form of a mathematical equation (MER 1: mathematical equation of a signal in time domain) and a signal spectra (MER 2: graphical representations of a signal in frequency domain). The reciprocative interface feature of Reciprocative Dynamic Linking allows both the representations to get manipulated. Learners are not just able to see automatic variation occurring in the signal spectra when signal equation (MER 1) is manipulated but are also able to see the variation occurring in the signal mathematical equation when signal spectra (MER 2) is manipulated.

## Research questions and hypotheses

One of the objectives of the study was to assess the effect of Reciprocative Dynamic Linking on the development of learners’ Representational Competence. Development of Representational Competence was judged by assessing how learners understand semantics of each representation, how they understand which parts of the domain are represented, how they relate representations to each other, and how they translate between representations (Ainsworth 1999). The first research question assessing learners’ development of Representational Competence was

RQ1: How does Reciprocative Dynamic Linking affect learning of Representational Competence in interactive learning environments?

Apart from assessing effectiveness of Reciprocative Dynamic Linking, investigating how it supports learners’ cognitive processing especially germane cognitive load was also necessary to analyze learning effectiveness of this feature. Thus, another objective of the study was to analyze impact of Reciprocative Dynamic Linking on cognitive load of learners. The second research question aimed at assessing learners’ cognitive load in the presence of Reciprocative Dynamic Linking. The inclusion of Reciprocative Dynamic Linking was foreseen as an additional learning support to learners that would positively influence their germane cognitive load. In line with this, the second research question was

RQ2: How is learners’ cognitive load influenced by the presence of Reciprocative Dynamic Linking in interactive learning environments?

To answer these research questions, we used the following types of learning environments: (a) Simulation (SIM) and (b) Interactive Simulation with Reciprocative Dynamic Linking (SIM-RDL). SIM and SIM-RDL were both designed for the same content for the selected topic. While SIM-RDL was designed with Reciprocative Dynamic Linking to offer reciprocative interface, SIM was designed without Reciprocative Dynamic Linking. The development of representation competence was assessed with the help of an instrument for measuring “Representational Competence within Signals and Systems domain.” Germane cognitive load was measured using validated a self-reported cognitive load subjective rating scale. The details of the instruments used are given in Section 5.3 of this paper.

The context of this research study has been a course on Signals and Systems, which is a foundation course offered in the second year of Electrical Engineering programs. The topic of the study, “Representation of signals in time and frequency domains,” demands learning from multiple representations. This topic forms the core knowledge required for building up knowledge related to different transforms (such as Fourier and Laplace transforms) which are regarded as important and difficult topics as reported in Signals and Systems education research literature (Wage et al. 2005). Deeper understanding of time domain and frequency domain representations, as well as mathematical-graphical translations, is mandatory for understanding this topic. The translation of a signal to its multiple representations has been reported as a learning problem in this course (Fayyaz 2014); hence, it is an important topic to address. The learning material developed offered MERs of three kinds: (i) linking time domain graph and frequency domain graph, (ii) linking frequency domain graph and time domain mathematical expression, and (iii) linking two different dynamically linked mathematical representations. All the questions expected learners to select appropriate MER, relate given MERs, and construct new MERs.

In terms of learning outcomes, students are expected to comprehend various concepts from the course and also to apply/analyze them in a meaningful manner while attempting associated procedural tasks. Thus, the “Understand,” “Apply,” and “Analyze” cognitive levels as defined in the Revised Bloom’s Taxonomy (Krathwohl 2002) were emphasized in this study. Work on the Signals and Systems Concept Inventory (Wage et al. 2005) and the work reported by Hiebert and Lefevre (1986) have emphasized on the need to focus on “Conceptual and Procedural” knowledge types as well as on their co-existence. One of the objectives of engineering curriculum has been to develop conceptual and procedural knowledge as mutually supportive factors (Taraban et al. 2007). This further supports the need for catering these two knowledge types while forming hypotheses. Thus, for both the research questions, hypothesis was formed for two different categories of knowledge type and three cognitive levels as per two-dimensional taxonomy of educational objectives (Anderson and Krathwohl 2001).

Firstly, it was expected that students learning with SIM-RDL would learn better as compared to students learning with SIM due to development of more Representational Competence. Thus, the hypotheses for RQ1 were as follows:

- H1-A: Students learning with SIM-RDL will score higher as compared to students learning with SIM for Conceptual knowledge at the “Understand” level.
- H1-B: Students learning with SIM-RDL will score higher as compared to students learning with SIM for Conceptual knowledge at the “Apply” level.
- H1-C: Students learning with SIM-RDL will score higher as compared to students learning with SIM for Procedural knowledge at the “Apply/Analyze” level.

Secondly, it was hypothesized that Reciprocative Dynamic Linking will improve learning in SIM-RDL due to increase in germane cognitive load of learners assuming all other cognitive loads experienced by learners remained constant. Thus, the following hypotheses were formulated for the second research question:

- H2-A: Students learning with SIM-RDL experience higher germane cognitive load as compared to students learning with SIM for the “Understand” level tasks for “Conceptual” knowledge.
- H2-B: Students learning with SIM-RDL experience higher germane cognitive load as compared to students learning with SIM for the “Apply” level tasks for “Conceptual” knowledge.
- H2-C: Students learning with SIM-RDL experience higher germane cognitive load as compared to students learning with SIM for the “Apply/Analyze” level tasks for “Procedural” knowledge.

## Methods

### Participants and experimental design

Participants were students from second year of engineering from three different colleges affiliated to University of Mumbai (*N* = 24; 14 males and 10 females). The study was conducted using a two-group post-test-only experimental research design. Since students were not exposed to the contents of the learning material in any of the courses studied in the previous semesters, post-test-only design was found to be appropriate for the study. Participants were randomly assigned to one of the following two conditions: (a) Simulation (SIM group); *N* = 12 and (b) Interactive Simulation with Reciprocative Dynamic Linking (SIM-RDL); *N* = 12.

The participants were studying in the third semester of the program. The course Signals and Systems is offered in the fourth semester of the program. Thus, the participants had no prior knowledge about the content of the simulation and they were at par for their prior knowledge based on their academic structure. Additionally, the prior knowledge was checked by giving two questions to solve before the treatment. In these questions, students were asked to represent a given sinusoidal signal in frequency domain spectral representation. The first question was, “A sinusoidal signal is described *x*(*t*) = 4cos (20*πt* + *π*/6). Can you identify its amplitude, frequency and phase?” The second question in continuation with the first one was, “Show how the signal will look when it is represented in frequency domain (i.e., plotted as a function of frequency as its horizontal axis).” As all the participants were familiar with mathematical form of representation of sinusoids, as anticipated, all participants answered the first question correctly. However, none of the participants could answer the second question. This not only implied that all the participants were at par as far as their background knowledge was concerned, but it also confirmed all of them to be novice learners for a topic on frequency domain representation of sinusoids. Thus, the purpose of administrating these questions to ensure equivalence of learners in both groups and to confirm that none of the students had studied the topic prior to the treatment was fulfilled. This pre-test was conducted before students worked with the interactive simulations. Its purpose was to check if the two groups were equivalent on the prior knowledge of the topic. However the pre-test scores were not considered for analysis of students’ performance after they worked with the interactive simulations, i.e., only the post-test scores were considered for analysis of performance. Further, first year performance grade point score (on a scale of 10) was used to confirm the group equivalence. The data was checked for normality and other valid assumptions to decide suitability of parametric statistical tests for comparing means. An alpha level of 0.05 was used for all statistical tests. There was not a significant difference in these scores for SIM (*M* = 8.26, SD = 0.86) and SIM-RDL (*M* = 8.35, SD = 0.85) groups grade point. As the sample size was small, we conducted non-parametric test on the data. The Mann–Whitney *U* test, a non-parametric equivalent test of independent sample *t* test, was used for comparing means of the Representational Competence assessment test scores. As per the results obtained from Mann–Whitney *U* test, the means were found to be statistically equivalent (*U* = 51.000, *p* = 0.802). The average age of students was 20 years. Participants were familiar with the use of ICT tools in learning through other courses and labs in their curriculum.

### Treatment

**Fig. 2:**
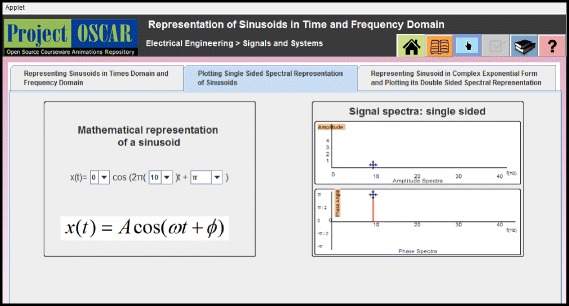
Screenshot of the Simulation applet developed on Signal representation

The instructional interventions for the two conditions were as follows:

- The SIM group learnt with an interactive JAVA applet. This applet allowed students to manipulate only one of the representations, and students could observe the changes happening in the second representation. The applet offered MERs of three kinds in three different tabs: (i) linking time domain graph and frequency domain graph, (ii) linking frequency domain graph and time domain mathematical expression, and (iii) linking two different dynamically linked mathematical representations. Figure 2 shows snapshot of the applet screen interface for tab 2 wherein time domain mathematical expression and frequency domain graphical representation are shown to learn time and frequency domains representations of sinusoids. Here, students could only manipulate mathematical representation of a sinusoid, and the provision of manipulating graphical representation of signal spectra was not offered to students from SIM group.
- Interactive Simulation with Reciprocative Dynamic Linking (SIM-RDL): This applet offered affordance in the form of Reciprocative Dynamic Linking. This Reciprocative Dynamic Linking offered interactivity that allowed learners to select and manipulate each of the MERs.

**Fig. 3:**
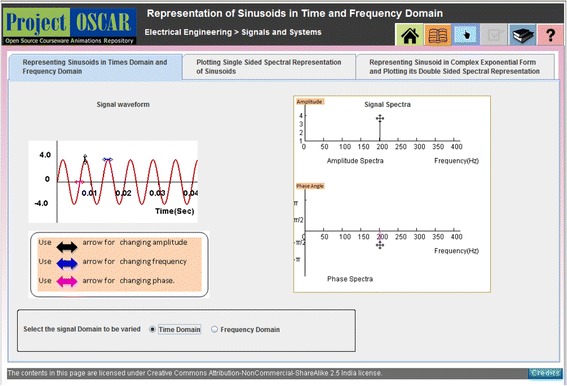
Screenshot of the SIM-RDL applet developed on Signal representation

Figure 3 shows two representations of a signal: time domain graphical representation and frequency domain graphical representation. The learner could select the representation to be manipulated. The reciprocative nature of dynamic linking allowed learners to vary time domain graphical representation and correspondingly observe dynamic changes happening in the frequency domain graphical representation of the signal, or vice versa. Similarly, in other tabs of the learning material, learners could implement reciprocative manipulation between either two different graphical representations or two different mathematical expressions using reciprocative interfaces.

### Measures and instruments

The following data sources were used:

1. Representational Competence assessment test
2. Self-reported cognitive load subjective rating scale
3. Recording of screen capture during learners’ interaction with learning material
4. Individual semi-structured interview (audio-taped and transcribed)

The learning impact of Reciprocative Dynamic Linking was analyzed from the scores of Representational Competence assessment test. The second research question was answered based on the self-reported cognitive load subjective rating scale. The screen capture analysis and individual semi-structured interviews were used to understand how students use Reciprocative Dynamic Linking affordance while learning. The qualitative data from screen captures and semi-structured interviews were analyzed initially to obtain a general sense of data as per six-step approach in analyzing and interpreting qualitative data (Creswell 2002, pp. 237).

#### Instrument for measuring Representational Competence within Signals and Systems domain

The instrument was designed to assess Representational Competence. It consisted of eleven questions, with ten open-ended questions and one question in a multiple choice format. Each of the questions expected students to carry out tasks that could assess development of Representational Competence; such as extracting information from the given representation, constructing new representations from previously learnt representations, evaluating consistency of different representations, integrating different representations to create a coherent understanding and to apply that to solve problems. The research context being a course of Signals and Systems, the questions from the instrument were set for one of the topics from the course. All questions required students to select, relate and construct multiple representations in the domain. The questions were related to (i) student’s understanding of the individual representation of signals, i.e., time domain/frequency domain representations or graphical representations/mathematical expressions, (ii) students’ ability to translate from one domain to another, i.e., from time domain to frequency domain and vice versa, and (iii) students’ comprehension of both representations in an integrated manner. Answering these questions required learners to use the given MERs in an integrated manner and further, to think on the underlying process/concept being depicted through these MERs. Thus, students’ abilities in answering these questions correctly reflected development of their Representational Competence. In the context of Signals and Systems, learners’ abilities to comprehend signal representations in both domains (time and frequency), to translate smoothly between domains, and to apply the integrated representation of a signal in newer contexts are indications of deeper and complete learning process.

In the topic of signal representation, underlying concepts related to signal frequency, amplitude, phase, fundamental time period, and complementary nature of time and frequency domains constituted the conceptual knowledge. Translating signals from one domain to another or from one representation to another required certain steps to be carried out in a sequential and meaningful manner. This was an example of procedural knowledge. With regard to cognitive level of the task, questions related to the “Understand” cognitive level expected learners to identify or interpret a particular representation. At the “Apply” cognitive level, students were expected to use their fundamental understanding of signal attributes in multiple domains and their interrelations in different domains while translating given signals or representations into another. The “Analyze” level questions expected learners to methodically examine the given information, identify the aptness of the information, and then solve the given task using the relevant part of the information.

**Fig. 4:**
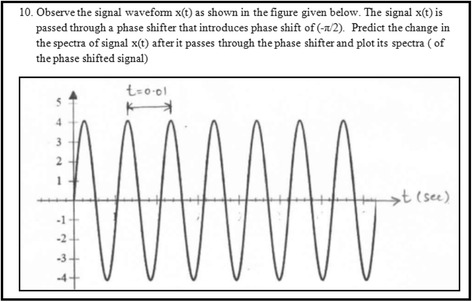
Screenshot of a sample assessment question

Three out of the ten open-ended questions were from an extended topic of Fourier transform properties. The learning material developed did not contain this topic. Also, students have not studied the topic in the previously learnt courses. These questions, apart from expecting students to translate from one representation to another, also expected them to analyze and translate their comprehension to an extended topic. These questions were from higher cognitive levels and helped in assessing students’ ability in integrating MERs and applying it to a new topic. One of the questions from the assessment instrument has been shown in Fig. 4, wherein, apart from expecting students to translate from one representation to another, it also expected students to analyze and translate their comprehension to an extended topic.

As the questions were set based on two-dimensional taxonomy of educational objectives (Anderson and Krathwohl 2001), they were categorized for specific type of knowledge and cognitive levels for the selected topic. It covered “Understand,” “Apply,” and “Analyze” cognitive levels and “Conceptual and Procedural” types of knowledge based on the two-dimensional taxonomy framework. The questions in the assessment test paper were organized into three categories based on their cognitive level, knowledge type, and MERs targeted. The category I questions catered to the “Apply Procedural knowledge” type, category II questions were from “Understand + Apply Conceptual knowledge” type, and category III questions aimed at “Analyze Procedural knowledge” type.

The answers of representational knowledge assessment test were assessed based on an adopted version of rubrics for assessing learner’s competency developed in selecting, constructing, and relating appropriate representation (Etkina et al. 2006: Revised and adapted based on https://sites.google.com/site/scientificabilities/rubrics). The rubric was designed to test six abilities: (i) ability to extract the information from the given representation correctly, (ii) ability to construct new representations from previous representations, (iii) ability to evaluate the consistency of different representations and modify them, (iv) ability to use/select appropriate representations to solve problems, (v) ability to represent mathematical expression (descriptive representation) sinusoidal/complex exponential, and (vi) ability to graphically represent (depictive) the form of signal waveform/spectra. The students were assessed on four levels of performance for these abilities. One of the abilities along with its performance indicators is shown in Table 1.

**Table 1:** Rubrics for assessing the open-ended questions from the instrument

Rubric for assessing learner’s competency developed in selecting, constructing, and relating appropriate representation Revised and adapted based on https://sites.google.com/site/scientificabilities/rubrics					
Ability		Missing	Inadequate	Needs improvement	Adequate
A2	Is able to construct new representations from previous representations	No attempt is made to construct a different representation.	Representations are attempted, but use of incorrect information or the representation does not agree with the information used. For example, showing double-sided/single-sided spectra in place of single-sided/double-sided spectra OR sinusoidal/complex exponential in place of complex exponential	Representations are created without mistakes, but there is information missing, i.e., units, labeling in the graphical representation	Representations are constructed with all given (or understood) information and contain no major flaws

##### Content validity by experts and interrater reliability

The Representational Competence assessment instrument was developed and peer-reviewed by the researchers of this study in cooperation with three domain experts who had +20 years of teaching experience in the domain of Signals and Systems. Two reviewers also had a formal background in educational technology research. The review process was carried out in an iterative manner. The suggestions given on time to time basis were incorporated, and the instrument was further reviewed till all the reviewers were satisfied with the categorization of the questions and their appropriateness in the context of learning objectives. A number of iterations were needed to finalize the test, especially for all the representations shown in the test paper. The instrument was also given to students (other than subjects of this study) to check its usability and language/diagrams comprehension. The questions were reworded wherever students expressed their difficulty in understanding the questions. Apart from this instrument, another instrument was used for measuring cognitive load.

Two raters assessed the answer sheets of Representational Competence assessment test independently, and the interrater reliability in terms of agreements was 95% for all open-ended questions from the instrument. The intraclass correlation coefficient was found to be 0.965. Disagreements were resolved via discussion between the two raters.

#### Instrument for measuring cognitive load

Apart from testing the hypothesis that “students would develop Representational Competence better with SIM-RDL as compared with SIM,” this research study also aimed at testing another hypothesis about the Reciprocative Dynamic Linking’s role in assisting learners by supporting germane cognitive load. Thus, another instrument needed in this research study was the one that could measure learners’ germane cognitive load. Cognitive load is a multidimensional construct representing the load that performing a particular task imposes on the learners' cognitive system. In an attempt to separately measure the three cognitive loads, it has been reported that mental effort ratings were most sensitive to manipulations of intrinsic processing (created by topic complexity), and mental difficulty ratings were most sensitive to indications of germane processing (reflected by transfer test performance) (DeLeeuw and Mayer 2008). These results were found to be consistent with a triarchic theory of cognitive load in which different aspects of cognitive load may be tapped by different measures of cognitive load. Learners have the ability to reflect on their cognitive processes and provide their responses on numerical scales (Gopher and Braune 1984; Paas et al. 2003). Therefore, self-reported measures were used to measure participants’ cognitive load. Uni-dimensional scales, such as retrospective difficulty ratings, are a popular subjective cognitive load measurement technique because they are easy to use and do not interfere with the learning task (Paas et al., 1994).

To measure mental difficulty indicative of germane cognitive load, a nine-point Likert-type scale was used as a subjective cognitive load measure. This scale is accepted as a valid method for measuring cognitive load (Kalyuga et al. 1998, 2000; Paas and Van Merrienboer 1994; Van Merriënboer et al. 2002; Yeung et al. 1997). In this study, participants were asked “How easy or difficult was to work with these questions?” after each category of questions. The participants selected one of the nine options: ranging from 1 as “extremely easy” to 9 as “extremely difficult.” A mental difficulty rating ranging from 1 to 9 was collected from each participant.

To measure intrinsic cognitive load, a subjective rating scale was provided on the first page of the students’ answer booklets. The participants were asked, “How much mental effort they invested while learning using the applets?,” and rated their subjectively experienced mental effort on a nine-point rating scale ranging from 1 “very very low mental effort” to 9 “very very high mental effort.” Nine-point rating scales have been used successfully in other studies (Kalyuga et al. 1998; Tindall-Ford et al. 1997).

### Procedure

#### Pilot study

The aim of the pilot study was to obtain insight into how students use the affordance of Reciprocative Dynamic Linking while learning from dynamically linked MERs. This insight was necessary to formulate a precise research problem to develop further hypotheses and subsequently test the learning benefits of the affordance. Considering the purpose of this research study, an “exploratory sequential mixed methods” design was found to be appropriate. The details of the pilot study have been reported (Patwardhan and Murthy 2015). The participants in this study were students from second year Electrical Engineering program studying a course on “Signals and Systems.” The participants belonged to two engineering colleges affiliated to University of Mumbai. A total of nine students (*N* = 9; female = 3, male = 6) participated in the study. In this study, the students interacted with the learning material with Reciprocative Dynamic Linking. Screen captures of the students’ interaction were recorded using CamStudio™ open source software. The screen captures were recorded for the entire time duration while the students interacted with the learning material. The students then solved open-ended questions assessing Representational Competence. After the assessment test, semi-structured interviews were conducted using interview protocol. The objective of the interview was to know the students’ perceptions about major issues like, “what kind of learning support did students get through Reciprocative Dynamic Linking” and “what aspect of learning could get influenced by Reciprocative Dynamic Linking?”

The pilot study was useful in confirming learning benefits of Reciprocative Dynamic Linking from qualitative aspect. The screen capture and interview data helped in understanding how students use the affordance of Reciprocative Dynamic Linking. The reciprocative interactivity helped learners in comprehending the representations in isolation, as well as the relation and translation between representations. This helped students in the development of Representational Competence. The granular translations shown in the assessment test was an evidence of the development of Representational Competence in students. This affordance was used by students to get support in the learning process that managed their cognitive resources optimally and also supported their inquiry process thus leading to deeper learning. A more interesting phenomenon observed in screen capture data was that learners returned again to the first representation after manipulating the second, that is, the confirmatory manipulation. We conjectured that while manipulating both representations, learners generated a hypothesis as part of mental inquiry process and returned to the first representation again to test or confirm the hypothesis. This conjecture was supported via interview data, wherein students reported that they used the feature that allowed variation in both graphs for checking how representations were related. In the following sections of the main study, its details and finding are presented.

#### Main research study

First, all participants were briefed about the study procedure and its objectives. They were assured that their participation had no bearing on their academic performance. After signing consent forms, they were allotted to two treatment conditions created using randomizer. The treatment intervention lasted for 35–40 min. During the treatment, screen capture was recorded using the CamStudio™ open source software to observe how students explore the interactive learning material. After completing learning from the respective learning material developed, participants were asked to solve the assessment test. The assessment test booklet had the following components: (i) Self-reported mental effort rating single-question questionnaire, (ii) Representational Competence assessment instrument for three different learning objectives, and (iii) Self-reported difficulty rating (mental load) single-question questionnaire. The assessment test format was arranged as follows:

- →Self-reported mental effort rating single-question questionnaire
- →Domain knowledge question of “Apply Procedural knowledge” --> Self-reported difficulty rating (mental load) single-question questionnaire for “Apply Procedural knowledge”
- →Domain knowledge question of “Understand and Apply Conceptual knowledge” --> Self-reported difficulty rating (mental load) single-question questionnaire for “Understand and Apply Conceptual knowledge”
- →Domain knowledge question of “Analyze Procedural knowledge” --> Self-reported difficulty rating (mental load) single-question questionnaire for “Analyze Procedural knowledge”

At the end of the research study, the students were interviewed. After the interview, they were thanked for their participation and were given participation certificate.

### Data analysis techniques

The quantitative data was collected in the form of Representational Competence assessment test score, self-reported mental difficulty score, and self-reported mental effort score for both groups. The Representational Competence assessment test score and self-reported mental effort score were designed for different categories of questions. Thus, the scores were compared for all these three categories independently. The questions in the assessment test paper were organized into three categories: category I questions catered to the “Apply Procedural knowledge” type, category II questions were from the “Understand + Apply Conceptual knowledge” type, and category III questions aimed at “Analyze Procedural knowledge” type. The second category of the questions was a mixed question category with questions of “Understand Conceptual knowledge” and “Apply conceptual knowledge” due to domain (time and frequency domains of a signal)-based categorization method adopted. This was done to maintain content coherence in the test.

Following steps were taken to carry out statistical analysis of data. The raw data was processed to get a normalized score, out of ten for each category of questions. The data was further checked for normality and other valid assumptions to decide suitability of parametric/non-parametric statistical tests for comparing means. An alpha level of 0.05 was used for all the statistical tests. The statistical analysis involved the following:

- Comparison of means of Representational Competence assessment test score to find out statistically significant difference between both groups using independent sample *t* test or its equivalent non-parametric test to test hypotheses H1-A, B, and C
- Comparison of means of self-reported mental difficulty score and self-reported mental effort score to find out statistically significant difference between both groups using independent sample *t* test or its equivalent non-parametric test to test hypotheses H2-A, B, and C

The qualitative data received from semi-structured interviews and screen captures were analyzed using Content Analysis method.

#### Screen capture analysis

The recorded screen captures were analyzed to find out the manner in which students explored the feature of Reciprocative Dynamic Linking offered by interactive learning environments. The screen captures were collected for all participants while they were interacting with the learning environment. As four captures were lost due to technical issue, total 20 screen captures were analyzed. Out of 20, 9 screen captures were for the control group (SIM), and 11 were for the experimental group (SIM-RDL). The time for exploring the material ranged from 7 to 20 min (average of 12:10 min). The objectives of screen capture analysis were as follows: (i) to identify the general approach of students while exploring the ILE, (ii) to analyze whether the IEF was used by the students, and (iii) to analyze pattern of exploration by both groups while using Reciprocative Dynamic Linking.

Considering these objectives, the qualitative analysis of screen capture was done in two phases. The phase I consisted of “code identification phase,” where the possible codes that could emerge were looked for and identified. In phase II, all the screen captures were analyzed again based on the identified codes. An exploration activity by a student was considered as a unit of analysis. For example, selecting a representation for manipulation by clicking on radio button, manipulating the selected representation, selecting the other representation for manipulation, and navigating between different tabs of learning material were some of the examples of various activities students did while exploring the content. While looking for general approach of exploration, the objective was to assess the exploration for any kind of abruptness in the navigation. Based on this, “structured navigation” or “non-structured navigation” was identified as codes in the first phase of analysis. The other objective for screen capture analysis was to identify if students used Reciprocative Dynamic Linking, which was coded under the category “utilization of affordance.” The third objective of screen capture analysis was to identify exploration pattern. The explanatory manipulation exploration and confirmatory manipulation exploration patterns were identified during pilot study. During this screen capture analysis, all the screen captures were analyzed to find out these exploration patterns.

#### Analysis of semi-structured interviews

All the twenty-four participants were interviewed face-to-face immediately after they completed the assessment test. The objective of conducting the semi-structured interview was to gather data about students’ learning experiences and their perceptions about the learning environment/its features.

Procedure: Students were briefed about the interview objective and protocol, and their consent for audio recording of the interview was taken. Then, they were asked about their learning experience. The conversation was based on the following open-ended questions: (1) Can you tell us something about the learning experience you had today? (2) Which typical aspect/feature of the learning environment you think must have helped you while learning? (3) In what way, you feel the learning environment features could help you while solving the domain knowledge assessment test? After asking about their own learning experience and the manner in which they utilized the learning environment for the purpose of learning, they were shown the learning environment of the other group, and their perception about it was asked ( i.e., control group participants were shown the experimental group learning environment and vice versa).

The interviews lasted for 8–10 min. The recorded interviews were transcribed and analyzed further using Content Analysis method with a “sentence” as the “coding unit.” The coding was done keeping in mind the objectives of the questions asked. Accordingly, the following categories of the codes emerged strongly from the analysis.

1. Learning pattern: This code elaborated the learning pattern followed by learner while learning the given content from the learning material.
2. Feature impact: This code focused on which feature of the learning material was perceived by learner to be useful in learning and how learners derived learning help from it.
3. Learning preferences: This code refers to the learning style/feature preferred by learners.

## Results

### Representational Competence assessment test

Table 2 shows the mean and standard deviations of Representational Competence assessment test scores for the research study. Both treatment groups were compared for Representational Competence assessed by questions set in three different categories. As mentioned earlier, these domain knowledge questions assessed development of learners’ Representational Competence in the topic of Signal Representation from a course on Signals and Systems.

**Table 2:** Mean scores and standard deviations of the Representational Competence assessment test score

Learning objectives	Representational Competence assessment test score			
	Simulation (SIM) *N* = 12		Interactive Simulation with RDL (SIM-RDL) *N* = 12	
	*M* (out of 10)	*SD*	*M* (out of 10)	SD
Category I (Apply Procedural knowledge)	4.48	2.16	6.20	1.94
Category II (Understand + Apply Conceptual knowledge)	6.37	1.18	7.11	1.34
Category III (Analyze Procedural knowledge)	5.17	2.65	8.44	1.99

The data presented in Table 2 passed Shapiro–Wilk test for normality, and other assumptions needed for parametric tests were found to be valid. However, as the sample size was small, we conducted non-parametric test on the data. The Mann–Whitney *U* test, a non-parametric equivalent test of independent sample *t* test, was used for comparing the means of the Representational Competence assessment test scores. As per the results obtained from the Mann–Whitney *U* test, the Representational Competence assessment test score means were found to be statistically significantly different for “category I: Apply procedural knowledge” and “category III: Analyze Procedural knowledge”: (*U* = 38.500, *p* = 0.043 and *U* = 16.000*, p* = 0.001, respectively). There was no statistically significant difference found in the means of “Understand + Apply Conceptual knowledge” category II questions scores (*U* = 41.000, *p* = 0.072).

We did a detailed analysis of questions from categories I to III to get more insight. The questions from category I aimed at assessing students’ ability of applying procedural knowledge while translating from one domain representation to another domain representation. Out of the five questions from category I, question numbers 2, 4, and 5 not just assessed students for the topic explained in the learning material, but the questions also covered the topics that could be treated as an extension of the topic. The better performance of SIM-RDL group students, especially in these questions, in a way indicated that the experimental group could develop deeper learning about the topic and was able to apply knowledge in different (unfamiliar) topics as well. The mean score of question numbers 2, 4, and 5 for SIM-RDL group was found to be 77.92% higher than the mean score for these questions for the SIM group. (The means for these questions (2, 4, and 5) together were found to be statistically significantly different after running Mann–Whitney *U* test *(U =* 31.500, *p* = 0.020). As far as category III questions were concerned, the question numbers 10 and 11 were from the “analyze” cognitive level. The score on these questions of SIM-RDL group was higher than that of SIM group by 63.15% (statistically significant means with *U* = 16.000, *p* = 0.001), and that again has been a supportive result. These findings demonstrated improved Representational Competence of SIM-RDL group learners as compared with SIM group learners measured by means of domain knowledge test questions.

### Self-reported difficulty level ratings

Table 3 shows self-reported difficulty level scores of learners. These scores are a measure of the germane cognitive load as experienced by learners while interacting with the learning environment.

**Table 3:** Mean scores and standard deviations of the cognitive load scores

Learning objectives	Self-reported difficulty level (germane cognitive load) scores			
	Simulation (SIM) *N* = 12		Interactive Simulation with RDL (SIM-RDL) *N* = 12	
	*M* (out of 10)	SD	*M* (out of 10)	SD
Category I (Apply Procedural knowledge)	5.58	1.24	4.27	1.27
Category II (Understand + Apply Conceptual knowledge)	5.25	1.71	4.55	1.73
Category III (Analyze Procedural knowledge)	6.08	1.68	4.36	2.06

The data presented in Table 3 passed the Shapiro–Wilk test for normality, and other assumptions needed for parametric tests were found to be valid. However, as the sample size was small, we conducted non-parametric test on the data. The Mann–Whitney *U* test, a non-parametric equivalent test of independent sample *t* test, was used for comparing the means of the self-reported difficulty level (germane cognitive load) scores. As per the results obtained from the Mann–Whitney *U* test, self-reported difficulty level (germane cognitive load) score means were found to be statistically significantly different for “category I: Apply Procedural knowledge” and “category III: Analyze Procedural knowledge”: (*U* = 29.500, *p* = 0.021 and *U* = 33.500, *p* = 0.041, respectively). There was no statistically significant difference found in the means of the “Understand + Apply Conceptual knowledge” category II question scores (*U* = 40.500*, p* = 0.106).

In addition to germane cognitive load, learners experience intrinsic and extrinsic cognitive loads while learning from ILEs. The assumption of equivalence of these two cognitive loads (i.e., intrinsic load and extrinsic load) across treatment groups was verified by controlling certain factors. The main factors considered for controlling intrinsic cognitive load were prior knowledge of learners, difficulty level and content of the topic to be studied, and academic characteristics of learners. Additionally, self-reported mental effort rating was used as a measure of intrinsic cognitive load to confirm the equivalence of intrinsic cognitive load in both groups (DeLeeuw and Mayer 2008). The learning materials for both groups were designed as per recommended instructional design practices to avoid extrinsic cognitive load. Also, equivalence of learning materials in terms of instructional design aspects, except presence or absence of Reciprocative Dynamic Linking, was verified. These points supported extrinsic cognitive load equivalence across groups.

The mean and standard deviation of learner’s self perception of “how much mental effort was invested while learning from SIM and SIM-RDL?” was found to be SIM (*M* = 3.92, SD = 1.16) and SIM-RDL (M = 4.20, SD = 1.75). There was no statistically significant difference reported in the means based on the findings of the Mann–Whitney *U* test (*U* = 52.500, *p* = 0.645). This mental effort reading being a measure of intrinsic cognitive load demonstrated that learners experienced same amount of intrinsic cognitive load while learning from two different treatment groups.

### Findings from screen capture analysis

**Fig. 5:**
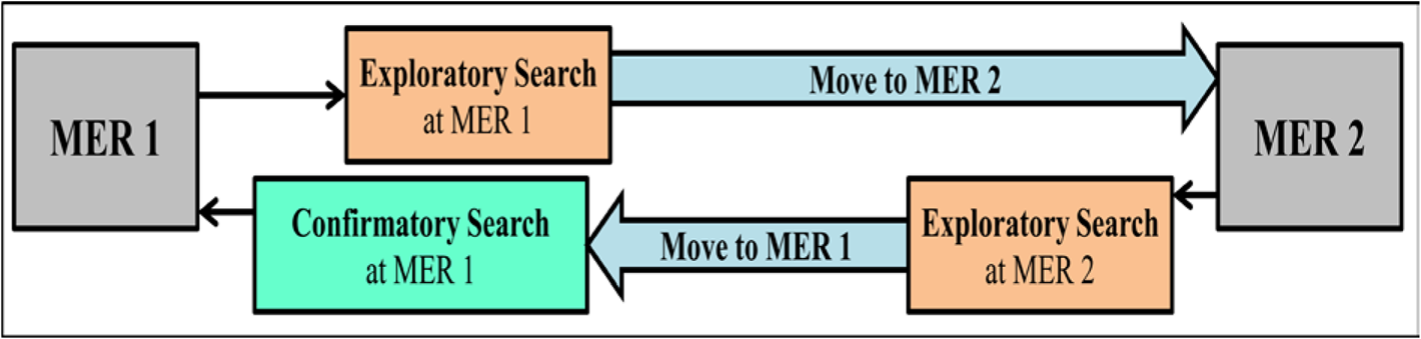
Screen capture analysis of navigation through learning material

As explained in Section 5.5.1, “General approach for exploring learning material,” “Use of Reciprocative Dynamic Linking affordances in SIM-RDL,” and “Exploration pattern” were identified as coding categories. The following have been the observations and inferences for the screen capture analysis for these three categories:

- General approach for exploring learning material (structured navigation/non-structured navigation): All participants exhibited structured navigation. The students moved linearly through “home screen --> theory --> learning content (tab wise) introductory text --> learning content interaction.” Students’ familiarity with computer-based learning environments/simulation environments was evident from this exploration approach.
- Use of Reciprocative Dynamic Linking affordances in SIM-RDL (used/not used): All students from SIM-RDL group used Reciprocative Dynamic Linking. That is, all of them manipulated both the MERs. Tab wise observations are as follows:
  Tab 1: Except for two, all students selected time domain for manipulation first. All possible variables were manipulated by students (amplitude, frequency, and phase) for both the MERs.
  Tab 2: All students selected both the MERs and manipulated all possible variables.
  Tab 3: All students selected both the MERs and manipulated all possible variables. Tab 3 also has a graphical representation, which was not offered for manipulation. Five students attempted to manipulate that and checked whether it was also offered for manipulation.
  SIM: All students from the SIM group used the possible variable manipulation opportunities offered for only one representation in all the three tabs.
  Thus, students from both groups fully utilized respective affordances offered in their learning materials. From domain perspective, time domain representation appears to be the more comfortable and familiar domain of representation and was preferred for manipulation as a first choice by maximum number of students. In general, all possible exploration opportunities and affordances were used by the students.
- Exploration pattern (explanatory manipulation exploration/confirmatory manipulation exploration of SIM-RDL): The pilot study revealed that many students followed confirmatory manipulation exploration pattern (Fig. 5). Similar observation was found in this phase of screen capture analysis. In a given tab, both representations were manipulated as confirmatory search.

While exploring MERs using Reciprocative Dynamic Linking, students manipulated the first MER, then the second one. After this, they again reverted to manipulate the first MER. This pattern was observed for all the three Tabs. This observation, resembling the “prediction and hypothesis testing phase” of inquiry cycle was confirmed in this screen capture analysis as well. This pattern was prominent in 9 students out of 11. In the case of SIM group, students used the offered affordances for more number of times. In an attempt to comprehend the content, students from SIM group probably had to use the only given affordance for many more number of times as compared to SIM-RDL group. Still, that could not translate into desired learning outcome, as was evident from the test score.

We present details about how screen capture analysis findings corroborated the findings from Representational Competence assessment test and self-reported difficulty level ratings in the Discussion section of this paper.

### Semi-structured interviews

Learning pattern, feature impact, and learning preferences were identified as codes while analyzing semi-structured interviews. While some of the verbatim responses corresponding to the identified codes have been presented here, the discussion related to the interview data is presented in the Discussion section.

Learning pattern:

*......“It’s basically when one of them moves, I like to observe this one is increasing and what’s happening to the next one, increasing or decreasing, that pattern I like to remember”........*

*..... Choosing anyone......so choose one and make changes over there see what changes happen in corresponding one then you can go for the second one...... make changes over there, then see.*

Feature impact:

*......“we are just back testing whatever changes we are seeing, are we are able to get the same changes mathematically back after changing this”......*

*.....“It works as a good rechecking for myself that if I have understood the concept like I can try to predict that if I move the right one in which direction or vice versa how it should work, so it’s a way of checking myself”.........*

*...... “with this, we will be able to find relations between all these.... it will simplify lot of things”.....*

*......“when one changes, the other has effects on it......... it creates a a..... like chain when more representations are there”.....*

Learning preferences:

*… “that would also be better because frequency domain …we can correlate frequency and time domain simultaneously, so if both go hand in hand then that--that would also be a better option and this helps the equation, like the equation we have to think about what will be the Sin or Cos Sin wave or the waveform”.......*

*....“if second changes and we need to find the changes in first then, uh, if the second option is selected then I will have to think it reverse, so it is difficult for me to you know think in other way. .... Okay......So if direct option is given to change in second and see the changes in first then that is obviously better”.*

*.....“if I understand, I do not need both ways manipulation.....one is also enough and sufficient”....*

The overall responses from interviews were in favor of Reciprocative Dynamic Linking. The important keywords/phrases emerged from the interviews favoring Reciprocative Dynamic Linking were *back testing*, *more flexibility in understanding*, *good rechecking*, *predict what can happen*, *can grasp in easier way*, able to find relations, *chain of representations*, *it fits in my mind*, *clear idea*, *relate better*, *help in thinking backwards*, and *time and frequency domain go hand-in-hand*.

During interviews, students demanded some additional features/content, such as more examples and audio commentary. Regarding learning preference, all 24 students advocated the need of Reciprocative Dynamic Linking. Students who learnt without Reciprocative Dynamic Linking explained what Reciprocative Dynamic Linking would be and how this feature could be added in the learning material that they had already used. After understanding about Reciprocative Dynamic Linking, all of them commented that they would have preferred learning from Reciprocative Dynamic Linking learning material and justified the reasons for their preferences using the keywords as mentioned above.

## Discussion

### Impact of Reciprocative Dynamic Linking on Representational Competence

The Mann–Whitney *U* test on the Representational Competence assessment test score assessing Representational Competence demonstrated that SIM-RDL group scored higher as compared to SIM group for category I and category III questions. The *p* values were found to be 0.043 and 0.001, respectively. These statistically significant *p* values implied that the means of test scores for questions related to the “Apply Procedural knowledge” and “Analyze Procedural knowledge” categories for SIM-RDL group were higher than those for SIM group. This confirmed the effectiveness of SIM-RDL group over SIM group. This also further confirmed that Reciprocative Dynamic Linking improved development of Representational Competence while attempting “Apply and Analyze Procedural knowledge task.” This supported hypothesis H1-C.

Category II had mixed questions at the “Understand Conceptual knowledge” and “Apply Conceptual knowledge” to fulfill domain-specific requirement covering both time and frequency domains representation of signals. When analyzed separately, the test scores were found to be statistically equivalent for the “Understand Conceptual knowledge” and “Apply Conceptual knowledge” task (*p* = 0.072). This statistically non-significant *p* value indicated equivalence of test score means of questions related to category II for both groups. This implied that Reciprocative Dynamic Linking could not offer significant help to learners to improve their scores in these two categories. The hypotheses H1-A and H1-B were not supported. We discuss about the probable reasons for this while answering RQ2 in Section 7.2.

The quantitative results when seen along with qualitative data collected from interviews and screen captures provide more insight to the inferences drawn. The physical interactions with MERs are a necessary part of one’s thinking process in the knowledge building process (Kirsh 2010). The feature of Reciprocative Dynamic Linking made this physical interaction with MERs possible. This was supported by the responses that emerged from the interviews of students. We restate some of the responses here: “more flexibility in understanding,” “can grasp in easier way,” “able to find relations,” “chain of representations,” “it fits in my mind,” “clear idea,” and “relate better*.*” These responses from students can be considered as an indication of the learning support that they could get from their interaction with Reciprocative Dynamic Linking. Additionally, due to the presence of Reciprocative Dynamic Linking, students could free up their cognitive resources and use them for developing better understanding of MERs, which got reflected in their higher test scores. The results related to cognitive load discussed in the next subsection also support this aspect.

The screen capture analysis revealed and confirmed the pattern followed by students while exploring the content. The students followed exploratory and confirmatory search pattern. The pattern suggested that the students were trying to check the mental model created through their interactions with Reciprocative Dynamic Linking. This again has been captured in some of the responses that emerged from the interviews: “back testing,” “good rechecking,” and “help in thinking backwards.” All these and similar responses articulated the process the students followed by interacting with Reciprocative Dynamic Linking. The reciprocative nature of the interaction was used by students first to build up the mental model of the content being learnt, and then, it was used to check the mental model created. The responses like “back testing,” “good rechecking,” and “help in thinking backwards” supported this. Science education literature associates prediction ability as one of the feature of model-making. The response, “I will be able to predict what can happen,” suggested that students could exhibit this ability as an outcome of model formation process. The better performance of students in the questions from extended topic in a way indicated that students could predict how the learnt knowledge would get applied in the new situations.

The main objective of Reciprocative Dynamic Linking was to develop students’ Representational Competence that would lead to improvement in learning. Here, the responses clearly articulated how students were able to develop Representational Competence as they were better equipped to “relate and link MERs” with Reciprocative Dynamic Linking. The responses such as “able to find relations,” “chain of representations,” “relate better,” and “time and frequency domains go hand-in-hand” supported this claim. Overall, learners who learnt with Reciprocative Dynamic Linking showed improvement in Representational Competence for category I and category III questions.

### Impact of Reciprocative Dynamic Linking on the cognitive processing of learner

The self-reported difficulty level ratings collected from learners have been found to be sensitive to indications of germane processing (DeLeeuw and Mayer 2008). Germane cognitive load enhances learning and results in task resources being devoted to schema acquisition and automation; it is a result of mental activities that are directly relevant to learning. The lower value of learners’ self-reported difficulty levels suggested that learning environment could offer more support to cognitive resources that directly contributed to the improvement in learners’ performance.

The Mann–Whitney *U* test on the self-reported difficulty level scores of learners revealed that the score means were found to be statistically significantly different for difficulty level reported for the “Apply and Analyze Procedural knowledge” category of questions (categories I and III *p* = 0.021 and *p* = 0.041, respectively). These statistically significant *p* values implied that the means of self-reported difficulty level scores for questions related to the “Apply Procedural knowledge” and “Analyze Procedural knowledge” categories for SIM-RDL group were lower than those for SIM group. This indicated that learners experienced higher germane cognitive load (less difficulty level) while learning with SIM-RDL as compared with the SIM group for the “Apply and Analyze Procedural knowledge” type of task. This supported hypothesis H2-C.

There was no statistically significant difference found in the means of difficulty level rating reported for the category II questions (*p* = 0.106). This implied that for category II questions, SIM-RDL group learning material did not offer any help to learners in improving their germane cognitive load; both the learning materials were found to be equivalent as far as learners’ germane cognitive load was concerned. For category II questions, the statistical equivalence of mental difficulty, along with the statistically non-significant difference between test scores, was analyzed further. We looked at the questions and analyzed the kind of mental efforts needed to put in for solving these questions. The questions from this category involved concepts related to signal frequency, amplitude, phase, fundamental time period, and complementary nature of time and frequency domains. Equal performance of students from both groups for these questions in a way suggested that both SIM and SIM-RDL offered equal learning support to learners while answering these questions. Learners perhaps needed no additional support for learning these basic concepts from the topic. As a result, learners might have felt the presence of Reciprocative Dynamic Linking in SIM-RDL redundant while catering to these questions. One of the verbatim responses was supportive of this (.....“if I understand, I do not need both ways manipulation.....one is also enough and sufficient”…). Some questions for which Reciprocative Dynamic Linking could not do any improvement in the learning scores expected learners to identify a representation and to write down mathematical expressions. These questions did not expect learners to construct new representations. Probably, students could solve these questions without need of any additional support. Students could perform better only with the help from Reciprocative Dynamic Linking in the questions that expected them to construct new representations. The very reason of introducing Reciprocative Dynamic Linking in the learning environment has been to support learners’ cognitive requirements that would help in the development of Representational Competence. It was hypothesized that learners demanded cognitive support while learning some types of tasks. Features like Reciprocative Dynamic Linking could offer such support, thus allowing some of the cognitive resources to get freed up to use while learning actual educational content.

To summarize, learners exhibited need for additional support in the form of Reciprocative Dynamic Linking for the development of Representational Competence while attempting tasks that required them to interpret each representation independently, relate multiple representations, and construct new representations based on the knowledge developed. This finding also indicated that Reciprocative Dynamic Linking led to improvement in Representational Competence owing to the increased germane cognitive load of learners. MERs have been widely used in science, technology, engineering, and mathematics (STEM) learning, and their learning potential has been widely accepted. Nevertheless, what technological and pedagogical affordances should be offered to learners in MER-based learning environments to facilitate learning have been still a widely explored research issue (Wu and Puntambekar 2012; Kozma 2003; Bodemer et al. 2004). The authors (Wu and Puntambekar 2012) have suggested various affordances such as dynamic linking, model progression, and sequencing as scaffoldings to assist learners while learning from MERs. The positive effect of an affordance of structured interaction while learning with different representations was reported by the authors (Bodemer et al. 2004). Irrespective of the precise nature of the affordances or scaffold designed in MER-based learning environments as suggested in these articles, the key point highlighted has been the need to assist learners while deriving learning potential of MERs. In this paper, working on the similar lines, the effect of the affordance of Reciprocative Dynamic Linking designed to assist cognitive processing of learners has been presented. The positive learning impact of Reciprocative Dynamic Linking as observed in the presented study emphasized the need to focus on features that recognize and fulfill cognitive demands of learners in technology-enhanced learning (TEL) environments.

### Limitations of the study

The small sample size is one limitation of this study. The logistics issues related to availability of students during pre-planned academic activities constrained the sample size. However, a serious attempt was made to triangulate the data from multiple sources. The inferences were drawn not only from the quantitative analysis but also from detailed qualitative analysis of screen-captured data and interviews. Even in the quantitative analysis, the test results were not based upon binary decision of correct/incorrect, but the assessment was done as per validated rubrics. The screen capture analysis revealed important exploration pattern of learners, and the interview analysis confirmed the inferences drawn from the analysis of screen captures. Adding more subjects to the sample size could further improve level of confidence about the contribution of results. Another limitation is that learners’ specific characteristics have not been considered as variable in the study. This was due to constraints from educational setup and the need to accommodate a variety of learners in the same educational setup. The third limitation is that, the study has been conducted in a single domain. Confirming the learning benefits of Reciprocative Dynamic Linking in another associated domain would further help in establishing generalizability. Another limitation may be the small duration of the treatment. The treatment intervention was for 35–40 min. However, the duration was found to be sufficient to learn the topic presented in the simulation. During pilot study, as well as during the main study, none of the participants demanded more time, neither while learning from simulation, nor while answering questions. From this observation, we decided that the learners spent sufficient time as was needed by their cognitive requirement to learn the content.

## Conclusions

The study was set with the objectives to investigate the role of “Reciprocative Dynamic Linking in developing students” Representational Competence and to investigate its effect on students’ learning in an interactive learning environment. Additionally, it also analyzed impact of Reciprocative Dynamic Linking on cognitive load of learners. The results confirmed that Reciprocative Dynamic Linking contributed in the development of Representational Competence as needed for higher cognitive level tasks. It also attributed development of Representational Competence to increase in germane cognitive load of learners.

The main contribution of this study has been the newly designed feature of Reciprocative Dynamic Linking to improve Representational Competence while learning from multiple external representations in interactive learning environments. Additionally, the linkage between learning effectiveness of this feature and learners’ germane cognitive load as established by the findings of this study could be considered as one of the means to create learning environments that optimize learners’ cognitive resources. With popularity of MERs in today’s computer-based educational content, developing effective learning environment that would match learners’ learning requirement is important. The rationale behind proposing and designing the Reciprocative Dynamic Linking was a blend of technology affordances, contemporary theories of cognition, and pedagogical requirement of the domain. The learning success of Reciprocative Dynamic Linking thus also advocates the need for such an approach while designing educationally effective learning environments.

Extending this study for some more topics from the same domain and from other relevant domains in future will help in validating its findings further. Incorporating more advanced research in the field of cognitive science in the study to assess cognitive constructs will bring more insight to the role of cognitive processing while learning from interactive learning environments.
